# *Candida albicans* Cells Lacking AP‐2 Have Defective Hyphae and Are Avirulent Despite Increased Host Uptake and Intracellular Proliferation in Macrophages

**DOI:** 10.1111/mmi.70032

**Published:** 2025-11-02

**Authors:** Stella Christou, Shannon Evans, Harriet Knafler, Iwona Smaczynska‐de Rooij, Kathryn R. Ayscough, Simon A. Johnston

**Affiliations:** ^1^ School of Biosciences University of Sheffield Sheffield United Kingdom; ^2^ School of Medicine and Population Health Florey Institute Bateson Centre University of Sheffield Sheffield UK

**Keywords:** *Candida albicans*, host pathogen interactions, medical mycology, phagocytosis, virulence mechanisms

## Abstract

*Candida albicans* is a commensal microbe and opportunistic human pathogen. *Candida* yeast are recognized and taken up by macrophages via phagocytosis. Macrophage surface receptors bind to specific components of the *Candida* cell wall. Following phagocytosis, *Candida* can respond to the host's intracellular environment by switching from a yeast to a hyphal morphology facilitating escape from macrophages and allowing subsequent invasion of host tissues. Various disruptions of *Candida*'s ability to form hyphae have been shown to reduce virulence and fitness in the host. Our previous work concluded that *Candida albicans* cells lacking AP‐2 (*apm4Δ/Δ)*, an endocytic adaptor complex, have increased cell wall chitin and morphologically defective hyphae in vitro. Increased chitin has been correlated with decreased recognition by macrophages, possibly due to masking of cell wall β‐glucan, the target for the Dectin‐1 immune receptor. Here we test the virulence profiles of *apm4Δ/Δ* mutant, demonstrating a surprising increase in macrophage phagocytosis that does not occur due to the elevated exposure of β‐glucan, highlighting the importance of cell wall components beyond chitin and glucan for macrophage engagement and uptake. Furthermore, the *apm4* mutant exhibited parasitism of macrophages, surviving and proliferating within the phagosome, a phenotype that was then replicated with a well‐characterized yeast locked mutant, demonstrating the further complexity of *C. albicans'* ability to evade macrophage responses. Finally, the combined phenotype of reduced hyphal formation but continued proliferation resulted in reduced virulence despite an equivalent burden of infection with wild‐type *Candida* infection, as determined using a zebrafish larval model of candidiasis.

## Graphical abstract

Mutation of exocytic cargo adapter *apm4Δ/Δ* results in reduced virulence of *Candida albicans* during infection despite promoting intracellular growth in macrophages.

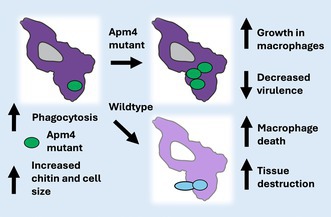

## 1 Introduction

*Candida albicans* is a commensal microbe and opportunistic pathogen of humans. Cellular innate immunity is essential for control of *Candida* infection and *C. albicans* cells are recognized and taken up by macrophages (Hernandez‐Chavez et al. 2017; Newman and Holly 2001; Scherer et al. 2020). The *C. albicans* cell wall is a complex polysaccharide structure that contains several chemical structures that are specifically recognized by the different immune cell receptors, for example, β‐glucan binding and recognition by Dectin‐1 (Gow et al. 2007; Hasim et al. 2017; Herre et al. 2004; Mansour et al. 2013). Increased levels of another cell wall component, the polysaccharide chitin lead to increased resistance to the anti‐fungal drug caspofungin and chitin appears to disrupt recognition of β‐glucan uptake by macrophages (Mora‐Montes et al. 2011). In addition to the molecular disruption of the immune response to infection, *Candida* can respond to the host environment by switching from a yeast to hyphal morphology, a strategy used to escape the macrophage phagosome (Vylkova et al. 2011; Vylkova and Lorenz 2014; Westman et al. 2018). Not only can the formation of hyphae support escape from macrophages but hyphal cells can also aid the invasion of host tissues (Moyes et al. 2016; Newman and Holly 2001; Seman et al. 2018; Swidergall 2019). Disruption of *C. albicans* cells' ability to form hyphae results in reduced virulence, survival and fitness in the host environment (Lo et al. 1997; Scherer et al. 2020; Seman et al. 2018).

We have previously demonstrated that deletion of the gene encoding the mu subunit (*apm4*) of the heterotetrametric AP‐2 endocytic adaptor complex in *C. albicans* leads to an inability to form a stable and functional endocytic complex at the plasma membrane. A major endocytic cargo that has been identified for *C. albicans* AP‐2 is Chs3, the major chitin synthase in *C. albicans* . The *apm4* deletion does not affect secretion of Chs3 but did lead to inhibition of its endocytosis resulting in elevated levels of Chs3 at the cell surface and increased levels of chitin in the cell wall (Knafler et al. 2019). Despite the cell wall changes, the *apm4∆/∆* strain can undergo a hyphal switch in appropriate conditions but the hyphae formed were shorter and wider. Heterozygous deletion of *chs3* in the *apm4∆/∆* strain, results in a reduction of chitin on the cell surface as expected and leads to a partial rescue of the hyphal morphology phenotype demonstrating the importance of chitin levels and its distribution for facilitating appropriate cell shape (Knafler et al. 2019). Given the phenotype of the *apm4* mutant we hypothesized that its virulence in macrophages and during invasive infection would be reduced due to its defect in formation of hyphae, but that increased cell wall chitin might enhance evasion of macrophages. Therefore, we sought to determine how the combined phenotypes of *apm4∆/∆* cells influenced the interaction of *Candida* with macrophages and the outcome of infection. Studying the interaction with macrophages, we revealed an unexpected increase in phagocytosis of *apm4∆/∆* mutant cells. Dissection of this phenotype revealed increased chitin did not reduce phagocytosis in this mutant and this was not due to compensatory elevated exposure of β‐glucan, highlighting the importance of cell wall components beyond chitin and glucan for macrophage engagement and uptake. Furthermore, despite its inability to form hyphae inside macrophages the *apm4* mutant still exhibited parasitism of macrophages, both surviving and proliferating within the phagosome, a phenotype that we then replicated with a well‐characterized yeast locked mutant, NRG1. Finally, using a zebrafish larval model of candidiasis we determined that the combined phenotype of reduced hyphal formation but growth within macrophages resulted in reduced virulence despite an equivalent burden of infection to wildtype *Candida* infection.

## 2 Results

### 2.1 Apm4 Deletion Results in Increased Uptake by Macrophages Despite Increased Chitin Levels but Similar β‐Glucan Exposure

**FIGURE 1:**
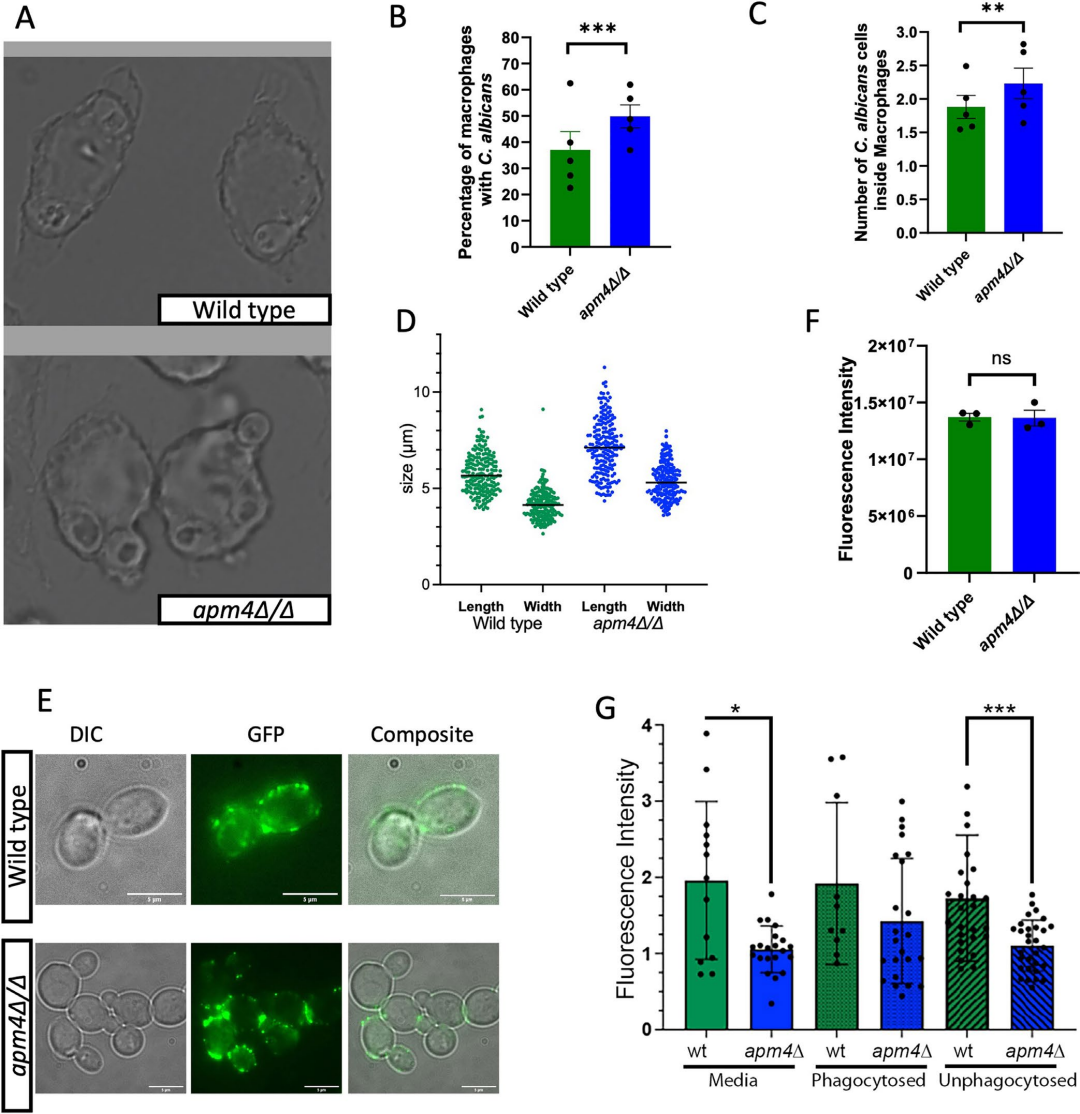
Deletion of *apm4* in *Candida albicans* results in increased levels of phagocytosis but exposed b‐glucan is unchanged. *C. albicans* and J774 macrophages were co‐incubated at 1:10 ratio for 30 min at 37°C, then fixed and imaged. (A) Maximum intensity projection images of Wildtype and *apm4Δ/Δ* infected macrophages. White arrows indicate phagocytosed *C. albicans* ; scale bar = 50 μm. (B) The proportion of macrophages in a field of view that phagocytose *C. albicans* were quantified. Five independent replicates with approximately six images per replicate. Macrophages were infected with a MOI 10 of *C. albicans* . Each circle represent the average of each replicate analyzed. Mann–Whitney statistical tests comparing the values of individual images per replicate. (C) The number of *C. albicans* per macrophage. Five independent replicates with 6 images per replicate. Circles represent the average number of *C. albicans* in macrophages per replicate. Mann–Whitney statistical tests comparing the values of individual images per replicate. (D) Overnight cultures of *C. albicans* were refreshed with YPD media for 30 min and then images of cells were captured and analyzed for cell size. *n* = 200 for each strain., *p* < 0.0001 for both length and width comparison between wildtype and mutant. The levels of exposed β‐glucan were investigated using immunofluorescence microscopy. (E) Overnight cultures of *C. albicans* were incubated with fresh media for 3 h before fixing and staining with Fc‐Dectin. (F) Fluorescence intensity of the cell staining was quantified as described. The graph represents the levels of exposed β glucan from three biological replicate analysed using a Mann–Whitney statistical test comparing the values of individual cells. (G) J774 macrophage‐like cells were infected with *Candida albicans* for 30 min before fixation, then stained and imaged for exposed Fc‐Dectin. The images captured were used to analyse levels of Fc‐Dectin in phagocytosed and non phagocytosed *C. albicans* and compare that to *C. albicans* cells that are incubated in media without J774 cells. The data represent the fluorescence intensity of the cells analysed using a Mann–Whitney test comparing the fluorescence intensity of individual cells. ns *p* < 0.05; *p = 0.05–0.01; **p = 0.01–0.001; ***p = 0.001–0.0001; ****p ≤ 0.0001.

Deletion of *apm4* has been shown by others to increase *Candida* cell wall chitin levels, measured by HPLC and immunofluorescence (Knafler et al. 2019) (Figure S1A). To determine whether the cell wall changes in the *apm4∆/∆* mutant influenced uptake by murine macrophages, adhered murine J774 macrophage‐like cells were infected with wildtype and *apm4∆/∆ C. albicans*. We found that the *apm4∆/∆ C. albicans* cells were phagocytosed by a greater proportion of macrophages (1.3‐fold increase; Figure 1A,B) and that each macrophage on average phagocytosed a larger number of yeast cells (Figure 1C). This finding indicated that either the increased chitin exposure did not reduce phagocytosis of *apm4∆/∆ C. albicans* or that there was an additional phenotype that was counteracting the effect of increased chitin in the cell wall during phagocytosis. We hypothesized two possible counteracting factors: that mutant cells were smaller which would facilitate more rapid phagocytosis or that β‐glucan levels were increased in *apm4∆/∆ C. albicans*. First, we measured the length and width of wildtype and mutant cells and found that the *apm4∆/∆ C. albicans* were in fact larger than wildtype cells (Figure 1D, Figure S1B). *C. albicans* are typically ellipsoid in their yeast form and this was unaltered in the *apm4∆/∆* mutant. Calculation of surface area based on length and width data indicated a surface area of 69 μm^2^ for wildtype cells compared to 111 μm^2^ for the mutant. Therefore, the *apm4*∆/∆ mutant presents a significantly greater surface area available for interaction with macrophages. Both total β‐glucan levels and its exposure at the cell surface are recognized as important factors affecting uptake of *C. albicans* cells by macrophages (Bain et al. 2014; Munro 2013). The total levels of β‐glucan in *apm4Δ/Δ* cells were previously reported to show no overall change compared to wildtype cells (Knafler et al. 2019). However, given the increased phagocytosis of mutant cells we investigated if there was greater surface exposure of β‐glucan and if this could be the factor responsible for the increased uptake and recognition by the macrophages (Davis et al. 2014; Galan‐Diez et al. 2010; Gow et al. 2007; Hasim et al. 2017; Mora‐Montes et al. 2011). For wildtype cells, the expected pattern of exposed β‐glucan in patches around the cell surface and near the bud neck regions was observed and this distribution was replicated in the absence of *apm4* (Figure 1E). Furthermore, there was no significant difference in the exposed β‐glucan levels between the two populations when fluorescence intensity of staining was quantified (Figure 1F). The results indicate that the changes in the cell wall composition of *apm4Δ/Δ* cells do not result in a change of the exposed β‐glucan levels and suggest that the increased phagocytosis is not linked to β‐glucan recognition. However, we also considered the possibility that β‐glucan levels could become altered when cells were exposed to mammalian cell media or in the presence of macrophages and that this resulted in increased exposure of β‐glucan on *apm4Δ/Δ* versus wildtype cells. To address this, the levels of exposed β‐glucan were measured during interactions with macrophages.

However, in contrast to our hypothesis the levels of exposed β‐glucan were decreased in extracellular *apm4Δ/Δ* cells in both media alone and in the presence of macrophages.

There was no difference, however, for either wildtype cells or *apm4Δ/Δ* when compared across all conditions (Figure 1G). The data indicated that as expected, the increased chitin in the *apm4Δ/Δ* mutant correlates with decreased exposure of β‐glucan. However, the increased chitin in *apm4∆/∆ C. albicans* did not lead to reduced uptake by macrophages and that factors other than β‐glucan and cell size are responsible for increased phagocytosis. In agreement with this, wildtype phagocytosis levels were partially restored in the *apm4* deletion with reduced chitin levels (Figure S1A) (Knafler et al. 2019). These experiments show that the phagocytosis phenotype was only partially rescued when the chitin levels are reduced (Figure S1C) suggesting that the phenotype observed is more complex than expected.

### 2.2 Intracellular *apm4∆/∆ C. albicans* Have a Defect in Hyphal Formation in Macrophages

**FIGURE 2:**
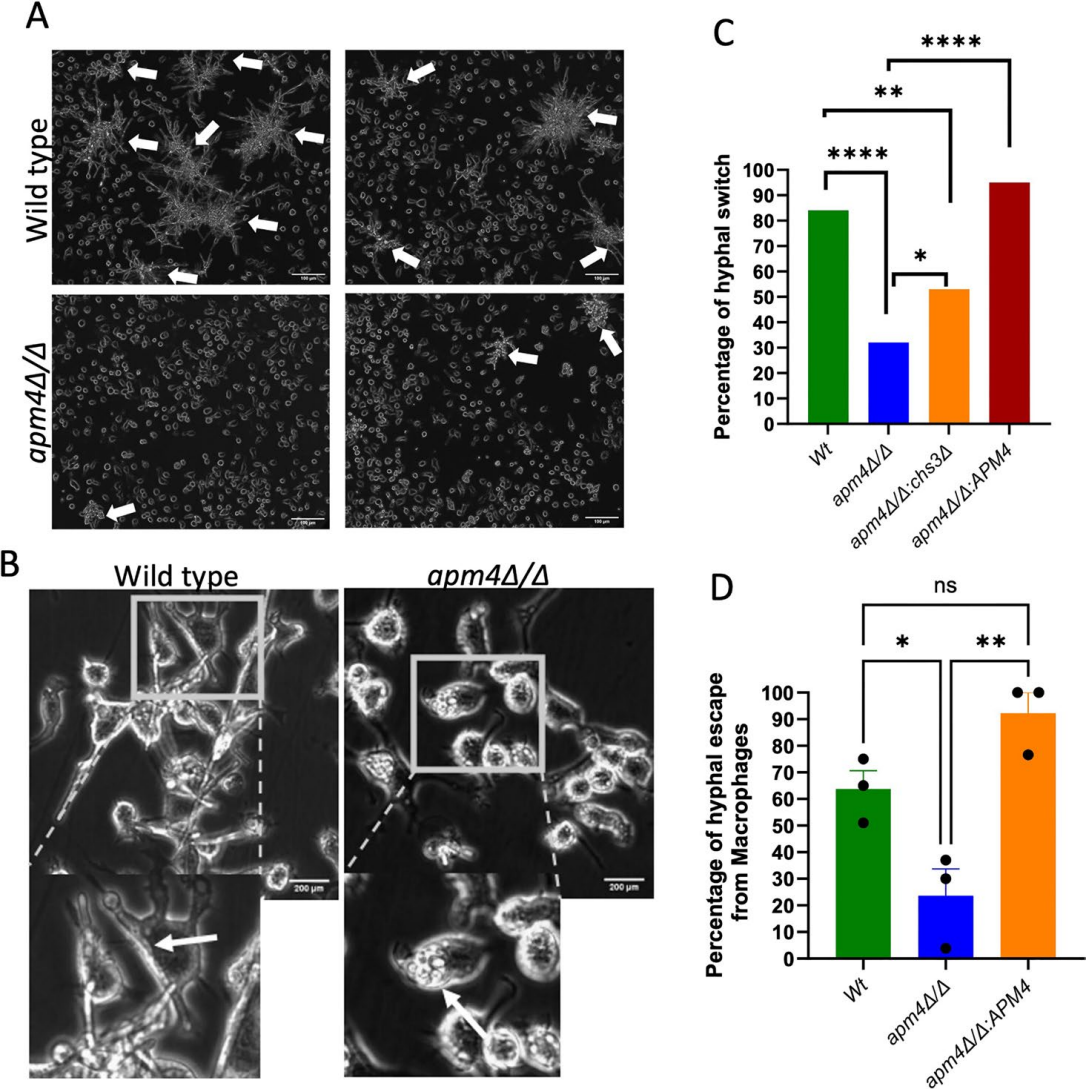
*Candida albicans* cells with an AP‐2 deletion show hyphal switch deficiencies. *C. albicans* and J774 macrophages at 1:1 ratio were co‐incubated for 18 h. The interacting cells were imaged at 20× and they were analyzed for hyphal morphology switching. (A) Representative images of infection after 18 h of interaction are shown. The white arrows indicate macrophage, *C. albicans* co‐clusters. Scale bars are 100 μm. (B) Time‐lapse captures at 10 h post interaction. The panels focus on individual macrophage cells, the white arrow illustrate phagocytosed *C. albicans* . (C) The time lapse images were analyzed by single cell observations for hyphal switch in the phagosome from approximately 75 infected macrophages from three biological replicates. The graph represents the quantification of hyphal switch inside the phagolysosome. Cell morphology changes that occurred only inside the phagolysosome were recorded. They results were analysed for each repeat via a Fisher's exact test. (D) The percentage of hyphae escaping host macrophages during an 18 h timelapse was analyzed via single cell observations of *C. albicans* cells in the phagolysosome from approximately 75 infected macrophages from three biological replicates. Analysis was performed using a one‐way ANOVA test for each biological repeat. ns *p* < 0.05; *p = 0.05–0.01; **p = 0.01–0.001; ***p = 0.001–0.0001; ****p ≤ 0.0001.

Following phagocytosis, wildtype *C. albicans* cells switch morphology, forming hyphae that can rupture the plasma membrane, lysing the host macrophage (El‐Kirat‐Chatel and Dufrene 2012; Hernandez‐Chavez et al. 2017; Westman et al. 2018, 2020). Our previous in vitro study of the *apm4∆/∆ C. albicans* demonstrated a defect in the formation of hyphae. Heterozygous deletion of the major chitin synthase *chs3* in the *apm4Δ/Δ* background, however, reduced cell wall chitin levels and partially rescued the ability of mutant cells to form true hyphae demonstrating an important and specific role for chitin in the hyphal deficiency phenotype (Knafler et al. 2019). We hypothesized that despite increased uptake into macrophages, intracellular *apm4∆/∆ C. albicans*, would be unlikely to lyse the cells due to the defect in hyphal switching. Using time‐lapse microscopy, we measured the formation of hyphae by intracellular *Candida* and the lysis of macrophages via single‐cell observation and quantification. While hyphal *Candida* were seen in nearly all fields of view following infection of macrophages with wildtype cells, they were rare following phagocytosis of *apm4∆/∆ C. albicans* (Figure 2A). To quantify this difference, we counted the number of yeast‐form (including budding cells) and hyphal‐ form (both true and pseudo‐hyphae) cell morphologies over 18 h of infection (Figure 2B). As expected, most intracellular wildtype *Candida* cells (> 80%) switched from a yeast to hyphal morphology over the 18 h of imaging (Figure 2C). In contrast, most *apm4Δ/Δ* cells remained in the yeast form. Hyphal‐like cells were observed in a proportion of macrophages infected with the *apm4* mutant, but no elongated hyphae or penetration of macrophages was observed, a phenotype that was rescued when the *apm4* gene was reinserted (~90%). Hyphal invasion of macrophages was quantified using single‐cell observations, and as expected infection with *apm4Δ/Δ* cells (20%) showed lower rupture of macrophages compared to wildtype cells (60%) (Figure 2D). We hypothesized that the *apm4∆/∆, chs3∆/CHS3* strain would be better able to form hyphae in macrophages, as had previously been demonstrated in vitro. In agreement with our hypothesis, we found that reduced chitin levels in this strain resulted in an increase in the proportion of yeast cells that underwent hyphal switching and invaded macrophages (Figure 2C,D). Thus, despite increased phagocytosis by macrophages and increased levels of cell wall chitin, the *apm4Δ/Δ* cells were less able to escape macrophages.

### 2.3 Intracellular Proliferation of Yeast Form Candida Cells in the Absence of Hypha Formation

**FIGURE 3:**
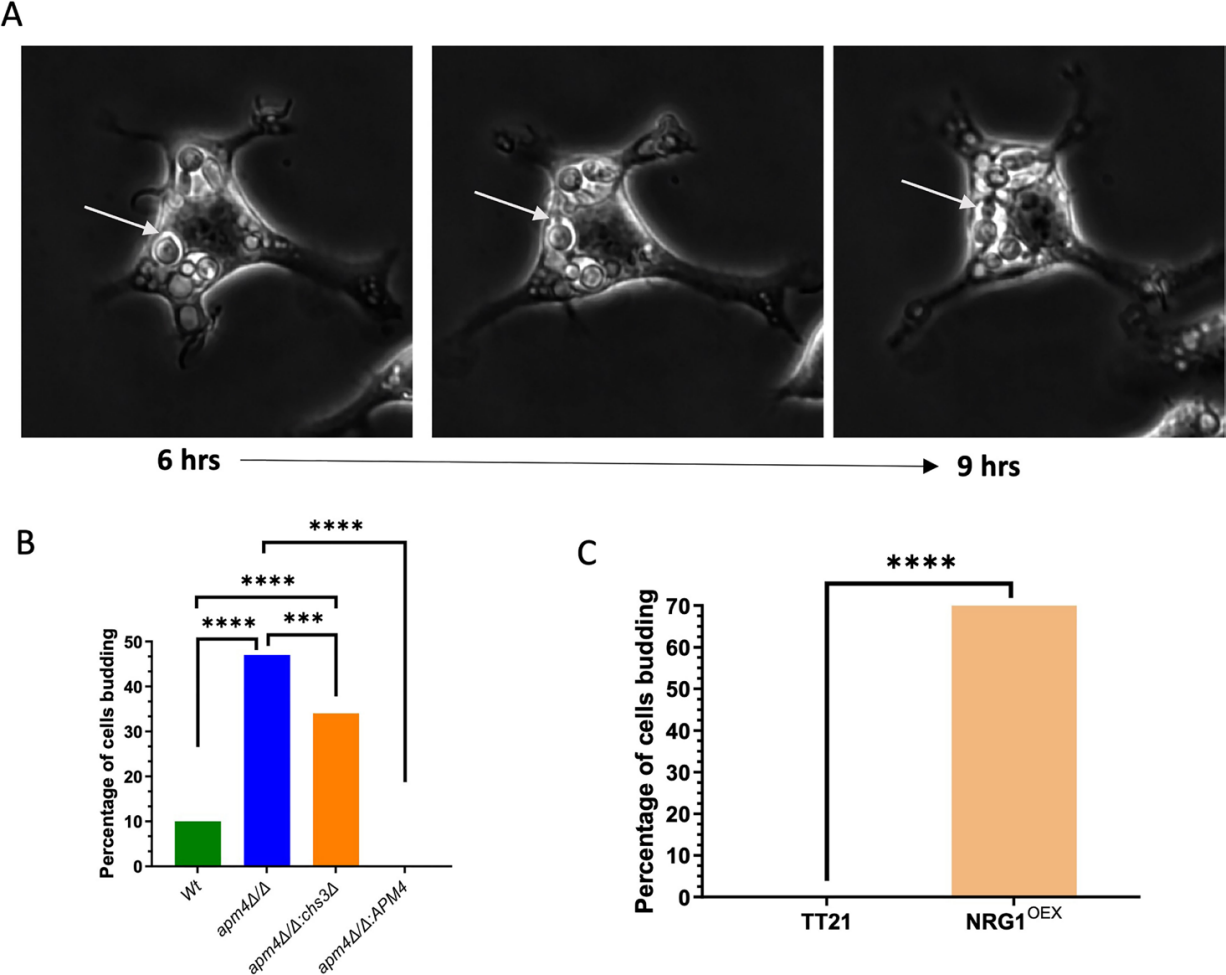
*Candida albicans* cells with *apm4* deletion grow inside the phagolysosome. *C. albicans* and J774 macrophages at a 1:1 ratio were co‐incubated for 18 h. The interaction was recorded via timelapse microscopy. Timelapses were then analyzed by single‐cell observations. (A) Timelapse images from the interaction of J774 cells with *C. albicans* over time. The white arrows show *C. albicans apm4Δ/Δ* cells budding inside the host. The data were analyzed for the percentage of cells forming buds inside the phagosome in (B) macrophages infected with wildtype, *apm4Δ/Δ* and *apm4Δ/Δ, chs3∆/CHS3* and (C) macrophages infected with TT21 and the yeast locked strain NRG1^OEX^. Approximately 75 macrophages from three independent biological replicates were analyzed. Fisher's exact test was used to analyze the statistical significance of the results. ns *p* < 0.05; *p = 0.05–0.01; **p = 0.01–0.001; ***p = 0.001–0.0001; ****p ≤ 0.0001.

Given the inability of *apm4∆/∆* mutant cells to lyse macrophages by the formation of hyphae, we predicted that macrophages would be able to control and digest intracellular *apm4∆/∆* cells. Using timelapse microscopy, we quantified the fate of intracellular *apm4∆/∆* mutant cells via single‐cell observation and quantification. Remarkably, we found that the mutant cells were not digested but instead were able to proliferate inside macrophages (Figure 3A,B). Over 40% of the *apm4∆/∆* cells that were phagocytosed were able to proliferate inside the phagosome; no proliferation was recorded in *apm4Δ/Δ:APM4* cells while fewer than 10% of the wildtype population exhibited this behavior. To determine whether proliferation was a switching response to the failure to maintain hyphal growth we tested the a*pm4Δ/Δ*, *chs3∆/+* cells. We predicted that the increase in hypha formation would proportionally reduce the cells that proliferated inside macrophages. In agreement with our prediction, we measured an almost identical reduction in budding compared to the increased rate of hyphal formation in a*pm4Δ/Δ*, *chs3∆/+* cells (Figure 3B). The proliferation phenotype is not directly linked to the metabolic activity measured as there was no significant difference recorded in NADH production during planktonic growth (Figure S1D). We next sought to establish whether the capacity to proliferate vegetatively in the phagosome was conserved in other mutants with a defect in the formation of hyphae. We chose the NRG1^oex^ mutant that does not form hyphae but through a different mechanism than *apm4∆/∆* cells and does not demonstrate any changes in chitin levels (Figure S1E). While *apm4∆/∆* mutant cells can initiate the formation of hyphae, their defect in endocytic recycling at the hyphal tip disrupts the progression of hyphal growth (Knafler et al. 2019), in contrast the NRG1^oex^ mutant is unable to initiate hyphal formation (Murad et al. 2001). The parental strain (TT21) formed hyphae in macrophages, and no budding cells were observed (Figure 3C). In contrast, the majority of NRG1^oex^ cells proliferated inside macrophages and at a greater proportion than the *apm4∆/∆* cells, as might be expected by the more severe hyphal switch defect in NRG1^oex^ cells. Therefore, we could demonstrate that two different *Candida* mutants with defects in hyphal formation were able to use alternative macrophage parasitism strategies and proliferate.

### 2.4 Impact of the *apm4* Deletion on Virulence in Zebrafish

**FIGURE 4:**
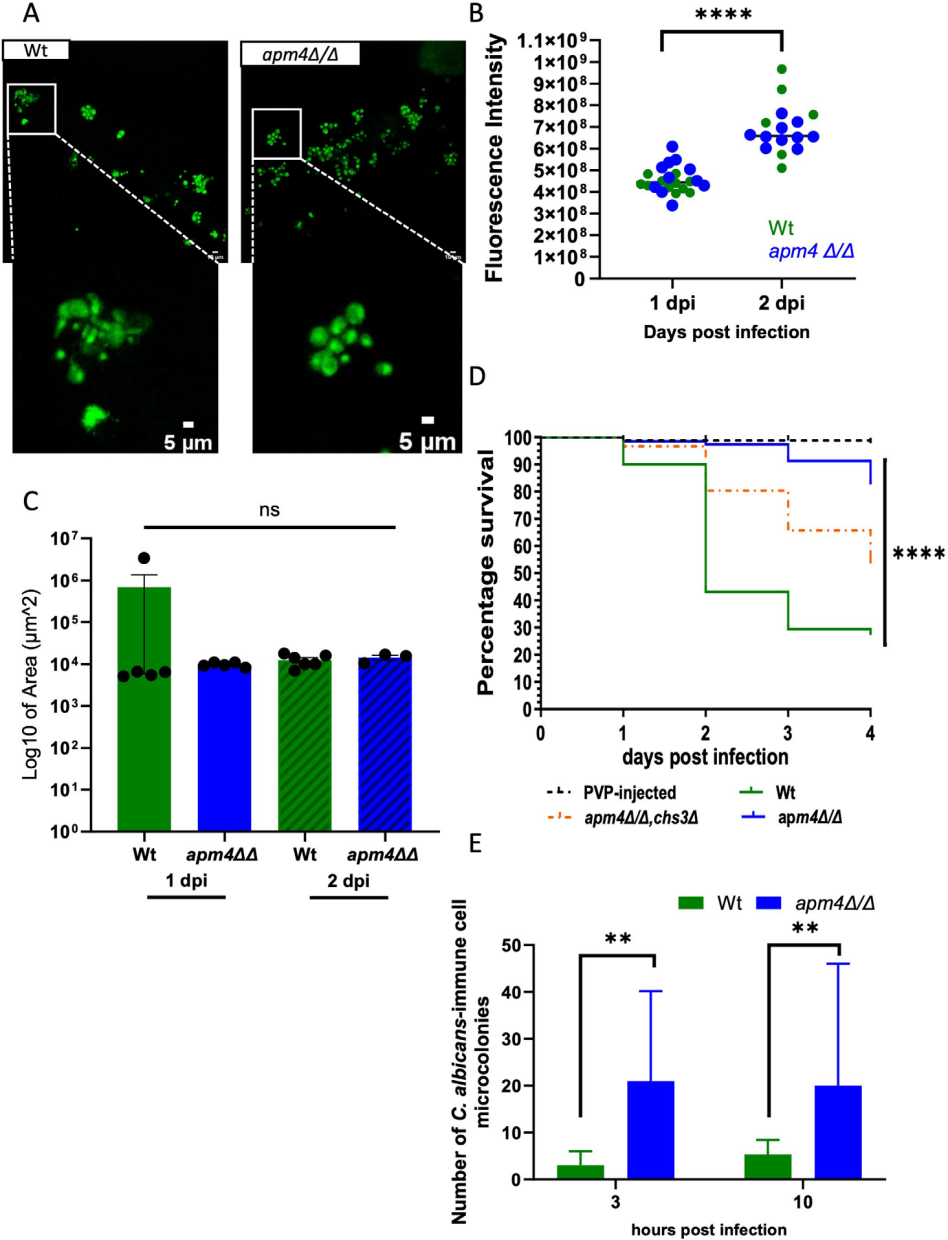
*Candida albicans* cells with *apm4* deletion have a hyphal switch deficiency during zebrafish embryo infections. Wildtype Nacre zebrafish embryos at 1 day post fertilization (dpf) were injected with 500 cfu of *C. albicans* in the caudal vein to monitor the infection progression. (A–C) (A) Confocal images of the infected embryos at 1 dpi were captured to observe the morphology of *C. albicans* cells in the zebrafish bloodstream. (B) The fungal burden of embryos infected was assessed using fluorescence from three biological replicates. Embryos were imaged at 1 and 2 dpi. Candida cells injected into embryos expressed cytoplasmic GFP, fluorescence emitted during microscopy represented Candida cells. The fluorescence intensity analysis was conducted using the Maximum Projection of Z stack images. (C) Embryos from three biological replicates were monitored for survival up to 5 dpf. Survival was assessed by cessation of heartbeat. The number of surviving embryos was counted every day until 4 dpi. The zebrafish were infected with the wildtype, apm4Δ/Δ, apm4Δ/Δ; chs3Δ and the PVP as an injection control. Survival statistical analysis concluded *p* < 0.0001 from an *n* = 90 embryos/group. KDRL mCherry zebrafish embryos with fluorescent vasculature were injected with 200 cfu of *C. albicans* in the caudal vein at 1 dpf (D, E). (D) Embryos from three biological replicates were fixed at 1‐ and 2‐day post infection and imaged the size of *C. albicans* microcolonies infecting the zebrafish embryos measured, six zebrafish per condition. (E) Zebrafish embryos with fluorescent blood vessels were infected with *C. albicans* imaged using fluorescent microscopy. Quantification is of *C. albicans* microcolonies that have disseminated from the blood vessels at 3‐ and 10‐h post infection. ns *p* < 0.05; *p = 0.05–0.01; **p = 0.01–0.001; ***p = 0.001–0.0001; ****p ≤ 0.0001.

The data thus far revealed an intriguing phenotype of *apm4∆/∆* cells in their interaction with macrophages. While unable to escape macrophages by the formation of hyphae, *apm4∆/∆* cells were able to still avoid killing and were observed to proliferate within macrophages. The decrease in the formation of hyphae in *C. albicans* is well evidenced to reduce its virulence in animal models of *Candida* infection (Lo et al. 1997; Seman et al. 2018). However, the balance of macrophage parasitism by intracellular proliferation versus cell and tissue damage by hyphae is less well understood. Therefore, we used a zebrafish larval model of *Candida* infection because we were able to directly visualize the *Candida* phenotype in vivo while simultaneously measuring the progression of infection. Bloodstream infection of wildtype Nacre strain zebrafish embryos 1 day post fertilization with wildtype *C. albicans* cells resulted in the presence of numerous hyphal *Candida* 1 day post infection (dpi) (Figure 4A). In contrast, as we had previously shown in vitro and following phagocytosis by macrophages, *apm4Δ/Δ* mutant cells were deficient in the formation of hyphae. We hypothesized that the *apm4Δ/Δ* mutant cells would proliferate in zebrafish in their yeast form as we had shown in macrophages in vitro. Comparison of fungal burden between wildtype and mutant cells was challenging because of the large difference in the populations of hyphal and yeast *Candida* between the two strains. Therefore, we used measurement of fungal burden in vivo in zebrafish using the fluorescence intensity of GFP‐tagged strains because this allowed the comparison of combined hyphal and yeast burden but it should be noted that fungal quantification by pixels suffers a bias against hyphal cells that are not as bright as yeast cells. We predicted that there would be no difference in fungal burden as the increased fluorescence of hyphal *Candida* would be matched by the increased number of yeast‐form *apm4Δ/Δ* mutant cells. Measurement of fungal burden 1 and 2 dpi demonstrated an increase over time but no difference in total fluorescence between wildtype and *apm4Δ/Δ* infections (Figure 4B). This hypothesis was further supported when we measured the size of *C. albicans* microcolonies inside zebrafish embryos, as the size of microcolonies in the live zebrafish embryos was similar for both populations (Figure 4C). Having demonstrated a similar phenotype of *apm4Δ/Δ* mutant cells in vivo in zebrafish in comparison to infection of macrophages, we sought to identify any differences in virulence between hyphal dominated wildtype infections and proliferating yeast dominated *apm4Δ/Δ* infections.

Infection of zebrafish embryos 1 day post fertilization resulted in 70% mortality over 4 days of infection. In contrast, very few *apm4Δ/Δ* infected zebrafish succumbed to infection and we found less than 20% mortality 4 days post infection (Figure 4D). Finally, using the intermediate phenotype *apm4 ∆/∆ chs3 +/∆* mutant we demonstrated increased virulence compared to *apm4 ∆/∆ C. albicans* further demonstrating the importance of chitin and hyphal formation for the virulence phenotype. Recent published work in zebrafish embryos suggested that *C. albicans* cells can use macrophages as a dissemination vector through the host (Scherer et al. 2020). We hypothesized that the *apm4Δ/Δ* cells would disseminate in the host as there was low mortality, but the fungal burden increased inside the host. Zebrafish embryos with fluorescent blood vessels (*Tg(kdrl*:*mCherry)s916*, Traver et al. 2003; Gibson et al. 2022) were infected in the caudal vein with *C. albicans* and it was followed by timelapse microscopy of infected embryos at 3 and 10 h post infection. The embryos were quantified for the number of *C. albicans* microcolonies that were outside the blood vessels. We reported that over time *C. albicans* microcolonies move from the bloodstream with initially 3% at 3 h and 5% at 10 h post infection for the wildtype cells. Dissemination from the bloodstream occurs more in zebrafish embryos infected with *apm4Δ/Δ* cells averaging 20% at both timepoints investigated (Figure 4E), supporting our findings that *apm4Δ/Δ C. albicans* exhibited an increased parasitic phenotype that resulted in increased dissemination in vivo.

## 3 Discussion

### 3.1 Phagocytosis Is Increased for *C. albicans* With an *apm4* Deletion

The cell wall of *Candida albicans* has long been recognized as a key component in the recognition and phagocytosis of the pathogen by macrophages. A significant body of research has demonstrated the importance of exposed β‐glucan in the cell wall binding to dectin‐1 receptors on macrophages as part of this uptake process (Davis et al. 2014; Galan‐Diez et al. 2010; Hasim et al. 2017; Mansour et al. 2013). Conversely, increased chitin has been considered a way by which *Candida* could mask β‐glucan and thus avoid immune recognition (Mora‐Montes et al. 2011; Munro 2013). In our previous work, we had shown that inhibition of the AP‐2 endocytic adaptor function through deletion of one subunit of the complex (*apm4*), led to an increased level of the major chitin synthase Chs3 at the *Candida* cell surface. There was a corresponding increase in cell wall chitin (Knafler et al. 2019). It was therefore unexpected in this study when the *apm4∆/∆* cells showed increased uptake by macrophages especially as the β‐glucan levels (studied via the Fc‐Dectin staining method) remained similarly exposed or slightly masked compared to wildtype cells. The results suggest that cell wall components other than β‐glucan are more important for the increased uptake of *apm4∆/∆* cells. Chitin currently has no known host receptors involved in its recognition, but it is thought to be co‐recognized with mannans (Netea et al. 2008; Snarr et al. 2017). Restoring the chitin levels of *apm4Δ/Δ* cells only partially rescues the phagocytosis phenotype. Previous analysis of the cell wall of *apm4∆/∆* cells revealed an increase in the thickness of the mannan layer albeit with no overall proportional increase in mannan (Knafler et al. 2019). Together the data support the idea that mannans are a critical component in recognition by macrophages and that recognition is not reduced in the *apm4∆/∆* mutant despite increased chitin levels.

### 3.2 The Virulence of *apm4* Null *C. albicans* Cells Is Reduced in Macrophages

Following uptake by macrophages, a *C. albicans* cell protects itself from host cell defense mechanisms and undergoes a morphological change to form a long projection or hypha to bring about host cell lysis (El‐Kirat‐Chatel and Dufrene 2012; Hernandez‐Chavez et al. 2017; Westman et al. 2018, 2020). The hyphal morphology is also critical for tissue invasion and damage through the production of the *C. albicans* toxin candidalysin (Moyes et al. 2016; Richardson et al. 2018). While in the macrophage, the pathogen induces host arginase to reduce the production of nitric oxide a key component in the host cell defense of murine macrophages; the host arginase response, however, is stronger during neutrophil interactions (Wagener et al. 2017; Burgess et al. 2023). We measured the hyphal switch of *apm4Δ/Δ* cells during interactions with the macrophage phagosome and found that *apm4Δ/Δ* cells have a hyphal switch deficiency, though many cells (~30%) do initiate short hyphae. Despite being observed within the phagolysosome, the mutant cells however did not appear to result in marked macrophage killing or invasion. Reduction of chitin in the cell wall by deletion of one copy of *chs3* in the *apm4* mutant led to a rescue in the proportion of cells that could form hyphae within the phagolysosome indicating the importance of chitin levels for the phenotypes observed. Virulence was also assessed during in vivo infection of zebrafish embryos where it was concluded that *C. albicans* cells with an *apm4* deletion are significantly less virulent than wildtype cells. *Candida albicans* strains that do not show a hyphal switch have previously been reported to be avirulent, with the yeast locked strains demonstrating this phenotype most clearly (Lo et al. 1997; Seman et al. 2018). A reduction in cell wall chitin in the mutant partially restored virulence reinforcing evidence for the importance of chitin in the formation of true hyphae and virulence (Lo et al. 1997; Seman et al. 2018).

### 3.3 Survival and Proliferation in the Host

As well as undergoing hyphal switch, another property of *Candida* cells following uptake by macrophages is that they induce host arginase and this acts to protect the pathogen from host defense killing mechanisms involving the production of nitric oxide (Wagener et al. 2017). *Candida* have been recently shown to have a host arginase response against zebrafish neutrophils (Burgess et al. 2023). This mechanism promotes *Candida* cell survival in the macrophage environment and has been considered a precursor to the formation of hyphae (Ghosh et al. 2009). In the case of the *apm4∆/∆* cells, however, which cannot produce lysis‐competent hyphae, we showed that cells continue to survive within the phagosome as budding cells were observed. This might indicate that the cells are able to induce host arginase irrespective of an ability to grow or maintain hyphae. Wagener and colleagues also demonstrated that increased surface chitin can lead to an increase in host arginase and that this can lead to pathogen protection (Wagener et al. 2017). Elevated chitin in the *apm4* mutant could therefore be postulated to be part of the mechanism facilitating mutant survival in the phagosome. This however cannot be the entire explanation for apm4∆/∆ survival as the continued budding of the yeast‐locked NRG1^oex^ cells in the same environment would suggest that increased chitin is not a prerequisite for continued proliferation (Figure S1E). We have made an initial investigation into whether metabolic changes are responsible for the observed proliferation phenotype. Our investigation via the measurement of NADH production in planktonic cells showed no significant difference between wildtype and proliferating cells (Figure S1D,F). However, this is only one method of assessing possible changes in fungal metabolism and future studies are needed to investigate this further. Our results suggest that *Candida*, irrespective of their capacity to produce hyphae, can induce conditions in the phagosome which facilitate its own survival and protect it from host killing mechanisms. While infection with wildtype *Candida* led to a high mortality, the *apm4∆/∆* mutant was able to continue to proliferate as judged by an increased fungal burden, but it did not lead to high levels of death. A similar finding has also been reported for the continued proliferation of yeast‐locked strains in zebrafish where it also concluded that macrophages can be used for dissemination to distal tissues (Scherer et al. 2020). In agreement with this, we found that *C. albicans* with an *apm4* deletion disseminated from the bloodstream more than wildtype cells demonstrating an alternative parasitic phenotype and proposing an alternative way for *C. albicans* pathogenicity. As in the case of macrophages, restoration of cell wall chitin to wildtype levels in the *chs3*∆ heterozygote (Figure 4), correlated with the capacity to form normal hyphae and partially restored virulence.

A potential limitation of our study is that we did not use complemented controls in all experiments. Hyphal formation and macrophage growth experiments were all performed with complemented strains, but our phagocytosis studies and zebrafish infection studies used strains that were rescued by single copy deletion of the AP2 cargo *chs3* as we had identified previously that this was sufficient to rescue the *apm4∆/∆* mutant phenotype (Knafler et al. 2019). Therefore, we should draw attention to this fact and that there may be other differences that might explain those particular phenotypes in our experiments.

Overall, our data indicate the complexities of host adaptation and evasion of *C. albicans* as a human commensal and opportunistic pathogen. The importance of an optimal level of cell wall chitin for the growth and maintenance of hyphae that are effective for macrophage lysis or tissue invasion in vivo is still to be established. Elevated chitin inhibited the formation of normal hyphae inside hosts but did not inhibit uptake by macrophages. The subsequent survival and proliferation of the *apm4∆/∆* mutant within macrophages and in zebrafish suggest a possible mechanism for non‐hyphal forming *Candida* to proliferate but remain avirulent within host organisms.

## 4 Experimental Procedures

### 4.1 Ethics Statement

Animal work was performed following UK law: Animal (Scientific Procedures) Act 1986, under Project License PPL 40/3574 and P1A4A7A5E. Ethical approval was granted by the University of Sheffield Local Ethical Review Panel.

### 4.2 Candida Culture and Strains

*C. albicans* yeast were grown overnight in YPD medium with 80 μg/mL L‐uridine. Overnight cultures were refreshed in new media for 30 min at 30°C before being used in any of the experiments performed in this study. Cells were washed three times in PBS, counted and used for infections as described for each experiment in the methods below. To allow imaging of live *Candida* cells without staining the cell wall cytoplasmic Eno1 was tagged with GFP using homologous recombination utilizing the auxotrophic markers of the SN148 strain (Noble and Johnson 2005). The deletion of both copies of the *apm4* gene was performed using the arginine and histidine auxotrophic marker while the Eno1 cytoplasmic tag was achieved via the leucine auxotrophic marker. The method used for genetic modifications in this study was used in previous studies by our group (Knafler et al. 2019) using lithium acetate. Strains were validated via colony PCR using external primers and sequencing. Tagged strains were analyzed to ensure they had the same phenotypes as untagged Wt and *apm4∆/*∆ cells published in our previous work. Strains and their descriptions are shown in Table 1.

**TABLE 1:** List and description of strains used in this study.

Strain name	Genotype	Origin
SN76 (KAF17)	*ura3Δ::imm* ^*434*^ */ura3Δ::imm* ^*434*^ , *iro1Δ::imm* ^*434*^ */iro1Δ::imm* ^*434*^, *his1Δ/his1Δ, arg4Δ/arg4Δ*	Noble and Johnson 2005
SN148 (KAF19)	*ura3Δ::imm* ^*434*^ */ura3Δ::imm* ^*434*^ , *iro1Δ::imm* ^*434*^ */iro1Δ::imm* ^*434*^, *his1Δ/his1Δ, arg4Δ/arg4Δ, leu2Δ/leu2Δ*	Noble and Johnson 2005
KAF 37	SN76 *apm4Δ*SN76 *apm4 Δ::HIS1/apm4Δ::ARG4*	Knafler et al. 2019
KAF 52	SN76 *apm4Δ::HIS1/apm4Δ::ARG4, CHS3/chs3Δ::SAT1*	Knafler et al. 2019
KAF 59	SN148 *apm4Δ::HIS1/apm4Δ::ARG4*	This study
KAF 62	SN148 ENO1/ENO1‐GFP::LEU2	This study
KAF 63	SN148 ENO1/ENO1‐GFP::LEU2, *apm4Δ::HIS1/apm4Δ::ARG4*	This study
KAF 95	SN148 ENO1/ENO1‐GFP::LEU2, *apm4Δ::HIS1/apm4Δ::ARG4, APM4::URA3* (integrated at the RPS1 locus)	This study
TT21	*ade2::hisG/ade2::hisG ura3::imm* ^*434*^ */ura3::imm* ^*434*^ *::URA3‐tetO‐* *ENO1/eno1::ENO1‐tetR‐ScHAP4AD‐3XHA‐ADE2*	Johnston et al. 2013
NRG1^OEX^	*ade2::hisG/ade2::hisG ura3::imm* ^*434*^ */ura3::imm* ^*434*^ *::URA3‐tet‐O‐NRG1* *ENO1/eno1::ENO1‐tetR‐ScHAP4AD‐3XHA‐ADE2*	Peters et al. 2014

### 4.3 Mammalian Cell Culture and Seeding for Infection

J774 murine macrophage‐like cells were maintained in DMEM with high glucose (Sigma) supplemented with 10% Fetal Bovine Serum, (Invitrogen) 1% 200mM L‐glutamine (Invitrogen), 1% Penicillin Streptomycin mix (Invitrogen) for up to 16 passages post‐thawing at 37°C 5% CO_2_. Confluent J774 cells were seeded at 5 × 10^5^ cells/mL density in 24‐well plates. On the day of the experiment, the cell health and confluency (approximately 80% confluency) were confirmed and the cell media was changed to serum‐free DMEM supplemented with 200 mM L‐glutamine and 1% Penicillin–Streptomycin mix.

### 4.4 *C. albicans* Immunostaining

Overnight cultures were incubated in fresh YPD medium for 30 min. After the incubation cells were collected and washed 3 times with PBS. For cell wall chitin staining the cells were resuspended in 1 mL PBS and 1 μL of a stock 1 mg/mL Calcofluor white (CFW) (Merck) was added to the suspension. The cells were incubated at room temperature for 5 min before being imaged with a 150× NA 1.45 NA objective on an IX‐81 inverted microscope (Olympus) with a Retiga R3 charge‐coupled‐device (CCD) camera (QImaging) and Micromanager software. For exposed β‐glucan staining after the incubation, cultures were centrifuged at 1000 rpm for 3 min, resuspended in 1 mL of 4% formaldehyde in PBS for 30 min and kept on ice. After fixation, cells were washed 3 times with PBS and resuspended in 5 μg/mL of Fc‐Dectin from a stock of 2 mg/mL for 1 h at 4°C (a kind gift from Prof. Gordon Brown, University of Exeter). As fixation with formaldehyde can alter cell wall structure, we checked that no difference was observed after live or fixed labelling (data not shown). Cells were washed with freshly made FACs buffer (0.5% Bovine Serum Albumin, 5 mM EDTA, 2 mM Sodium Azide in PBS) then resuspended in secondary antibody anti‐human IgG conjugated with Alexa‐Fluor 488 (Jackson Immuno Research Laboratories). The final concentration of the antibody was 1.25 μg/mL. Cells were incubated for 45 min on ice in the dark. Following incubation cells were washed three times with PBS before being imaged with a 150× NA 1.45 NA objective on an IX‐81 inverted microscope (Olympus) with a Retiga R3 charge‐coupled‐device (CCD) camera (QImaging) and Micromanager software (Bain et al. 2014).

### 4.5 Phagocytosis of *C. albicans*

A 1 × 10^5^ cells/mL suspension of J774 macrophages was seeded on a sterile glass coverslip inside a 24‐well plate and incubated overnight at 37°C 5% CO_2_ in DMEM with high glucose (Sigma) supplemented with 10% Fetal Bovine Serum, (Invitrogen) 1% 200mM L‐glutamine (Invitrogen), 1% penicillin streptomycin mix (Invitrogen). Before infection the media were changed to serum‐free DMEM. *Candida* overnight cultures were incubated for 30 min in fresh media before being washed and counted to create a *Candida* cell suspension of 1 × 10^6^ cells/mL. The macrophage media was changed to serum‐free media, and then they were infected at a 1:10 macrophage to *Candida* ratio. Cells were co‐incubated for 30 min at 37°C 5% CO_2_. After the incubation the cells were washed with PBS and fixed with 4% formaldehyde (Thermo Scientific) at room temperature for 10 min. Slides were washed with PBS and distilled water. The coverslip was attached to the glass slide using 6 μL of Mowiol mounting media (Sigma). The slides were imaged with a Nikon Eclipse Ti microscope using NIS Elements software (Nikon) with a Plan Apo 60× 1.4 NA DIC objective capturing a Z stack distance of 10 μm slicing at every 1 μm with an Andor NEO sCMOS camera. The DAPI channel was used to visualize the CFW stain of *C. albicans* and the macrophages were visualized using DIC contrast. The images were analyzed to determine the percentage of macrophages that contained *Candida* and the number of *C. albicans* cells per macrophage; this was achieved via analysis across the Z‐stack if the macrophage encapsulated the fungus to determine internalization.

### 4.6 Timelapse of Candida Macrophage Interactions

The microscope chamber was pre‐warmed to 37°C with 5% CO_2_, 80% confluency J774 cells on 24 well plates were prepared as previously described for infection and *C. albicans* cultures from overnights were refreshed for 30 min before being washed and counted. 5 × 10^4^ cells/mL *C. albicans* cells were used for infection and the timelapse experiment capture was started. Imaging was performed with a Nikon Eclipse Ti microscope using NIS Elements software (Nikon) with a ×20 Lambda Apo NA 0.75 phase contrast objective with an Andor NEO sCMOS camera. *C. albicans* cells were captured via the GFP channel and macrophages were visualized via the phase contrast channel for the periphery. The microscope captured images every 10 min for 18 h allowing for the study of the interactions. The timelapses were used for manual analysis of interaction phenotypes.

### 4.7 Exposed Dectin‐1 Levels During Macrophage Interactions

Macrophages were infected with *C. albicans* for 30 min before being fixed and stained. Macrophages were permeabilized with 40 μL per well of 0.25% Triton X 1000 for 3 min. After the permeabilization step the cells were washed 3 times with PBS and then the *C. albicans* immunofluorescence staining protocol above was followed. The cells were washed before imaging with a Nikon Eclipse Ti microscope using NIS Elements software (Nikon) with a Plan Apo 40× 1 NA water immersion lens with an Andor NEO sCMOS camera. The data from the images were then collected using the FIJI software by manually drawing around the *Candida* cell periphery to measure using the software the integrated intensity for each channel. The controls for these experiments were cells that were not incubated with macrophages and cells that were incubated with macrophages but not phagocytosed.

### 4.8 Zebrafish Survival and Fungal Burden

The zebrafish handling and injection was followed according to the method previously described by (Bojarczuk et al. 2016). 500 Colony forming units of washed overnight cultures of *C. albicans* were used to inject 1dpf Nacre embryos (Wildtype) in the caudal vein. The nacre strain is a wildtype strain of zebrafish that does possess melanocytes and is therefore superior for fluorescent imaging of infection and has been well validated for fungal infection studies (Bojarczuk et al. 2016; Duvenage et al. 2019; Evans et al. 2019; Gibson et al. 2022). The colony forming units were quantified via injecting into a 20 μL drop that was spread on a YPD agar plate and counted after incubation overnight at 28°C. The control for the injections was clear PVP + 20% Phenol red. Each injection group was composed of 30 embryos per replicate for three biological replicates. Zebrafish survival was monitored up to 4 dpi and the number of deaths was recorded every 24 h. The death of a zebrafish was determined by cessation of heartbeat. Survival curves were generated in Graphpad Prism for each replicate; statistical test, Mantel‐Cox survival test.

The fungal burden studied through fluorescence involved zebrafish infection with *C. albicans*; the embryos were imaged daily up to 3 dpi. The Nikon Eclipse Ti microscope was capturing Z stacks of live zebrafish embryos at 10× magnification as described [previously in detail (Bojarczuk et al. 2016)]. The maximum intensity projections of the Z stacks were analysed using the FIJI software by drawing around the perimeter of the zebrafish and quantifying the integrated intensity. It should be noted that fungal quantification by pixels suffers a bias against hyphal cells that are not as bright as yeast cells. The results were normalized to uninfected zebrafish from the same experiment. The results were analyzed in Graphpad Prism software using a one‐way ANOVA statistical test.

### 4.9 Fixing and Imaging Fixed Zebrafish Embryos

Nacre wildtype embryos or embryos with mCherry labelled vasculature *Tg(kdrl*:*mCherry)s916* (Traver et al. 2003; Gibson et al. 2022) were used to identify growth inside blood vessels. Zebrafish embryos at 1 dpf were injected with 200 cfu of *C. albicans* expressing cytoplasmic GFP (overnight cultures were grown in fresh media for 30 min before preparation for infection); as a control zebrafish were injected with PVP + 20% Phenol red. Infected zebrafish were maintained in a 28°C incubator. At 1 dpi about 10 zebrafish embryos per group were fixed in a 1.5 mL microcentrifuge tube with 4% formaldehyde (Thermo Scientific) overnight at 4°C. The next day formaldehyde was removed, and the embryos were washed twice for 2 min with PBS‐Tween (Sigma) (1:200 dilution of 20% Tween20). For the imaging step the fixed embryos were mounted on imaging plates and were imaged using a Plan Apo 40× 1 NA water immersion lens on a Nikon A1 confocal microscope using the mCherry detectors to image the vasculature of the KDRL embryos (*Tg(kdrl*:*mCherry)s916*, Traver et al. 2003; Gibson et al. 2022) and the GFP channel to image *C. albicans* to study the fungal burden or the Zeiss LightSheet Z1 microscope to study dissemination. A *C. albicans* and immune cell mass in the embryos was what we referred to as a microcolony. Microcolonies were quantified for size using the Arivis software. Microcolonies were identified in each infected embryo and were quantified for their area in μm^2^. Dissemination was assessed via the number of microcolonies that were identified outside of vasculature (as the zebrafish embryos were injected in the bloodstream) using infections of KDRL:mCherry embryos.

### 4.10 Quantification of Single Cell Observations and Image Analysis

The proliferation timelapse experiments were analyzed by observations of every macrophage and the *Candida* cells inside it throughout the course of the timelapse. Observations about how the *Candida* cells looked before uptake and the changes that occurred during the interaction were recorded. The timepoints of those changes were also recorded. The results were then processed and presented as proportions using Prism software. Only changes inside the phagolysosome were recorded. The Measure function in FIJI software was used for the quantification of fluorescence to assess different objects (cells) in images. Area, mean intensity, and integrated intensity were recorded. The process was manual as shapes were drawn around the periphery of cells to create a closed object.

### 4.11 XTT Assay

*C. albicans* from overnight cultures were counted and 1 × 10^4^ cells were plated in 96‐well plates with DMEM GlutaMax media (Gibco) + 15% Fetal Bovine Serum (Invitrogen). The cells were grown for 24 h at 37°C with 5% CO_2_ with no shaking before the XTT assay mix (Cayman Chemical assay kit) was added to each well. The XTT assay was incubated at 37°C with 5% CO_2_ for 4 h before imaging using a plate reader (ClarioStar, BMG) for absorbance at 450 nm. The experiment was repeated three independent times with three technical replicates per assay plate.

### 4.12 Statistical Analysis

The statistical tests used in this study were a Mann–Whitney test, Fisher's exact test, one‐way ANOVA, and a Mantel‐Cox survival test, unless otherwise stated. The stars in figures represented the significance values with a confidence interval of 95%. No significance was illustrated by ns *p* < 0.05; *p = 0.05–0.01; **p = 0.01–0.001; ***p = 0.001–0.0001; ****p ≤ 0.0001. GraphPad Prism version 10 was used for all the results presented.

## Author Contributions

**Stella Christou:** conceptualization, investigation, writing – original draft, methodology, visualization, writing – review and editing, resources. **Shannon Evans:** investigation. **Harriet Knafler:** investigation. **Iwona Smaczynska‐de Rooij:** investigation. **Kathryn R. Ayscough:** conceptualization, funding acquisition, writing – original draft, methodology, writing – review and editing, supervision, resources. **Simon A. Johnston:** conceptualization, writing – original draft, methodology, writing – review and editing, supervision, resources.

## Supporting information


**Figure S1:** mmi70032‐sup‐0001‐FigureS1.docx.

## Data Availability

The data that support the findings of this study are available from the corresponding author upon reasonable request.
