# Supplementary material for: Candida albicans Cells Lacking AP‐2 Have Defective Hyphae and Are Avirulent Despite Increased Host Uptake and Intracellular Proliferation in Macrophages
Source: Mol Microbiol. 2025 Nov 2;125(1):1–12. doi: 10.1111/mmi.70032 (PMC12763539; doi:10.1111/mmi.70032)
Supplement: Supplementary file 1 — Figure S1: mmi70032‐sup‐0001‐FigureS1.docx. [file MMI-125-1-s001.docx]

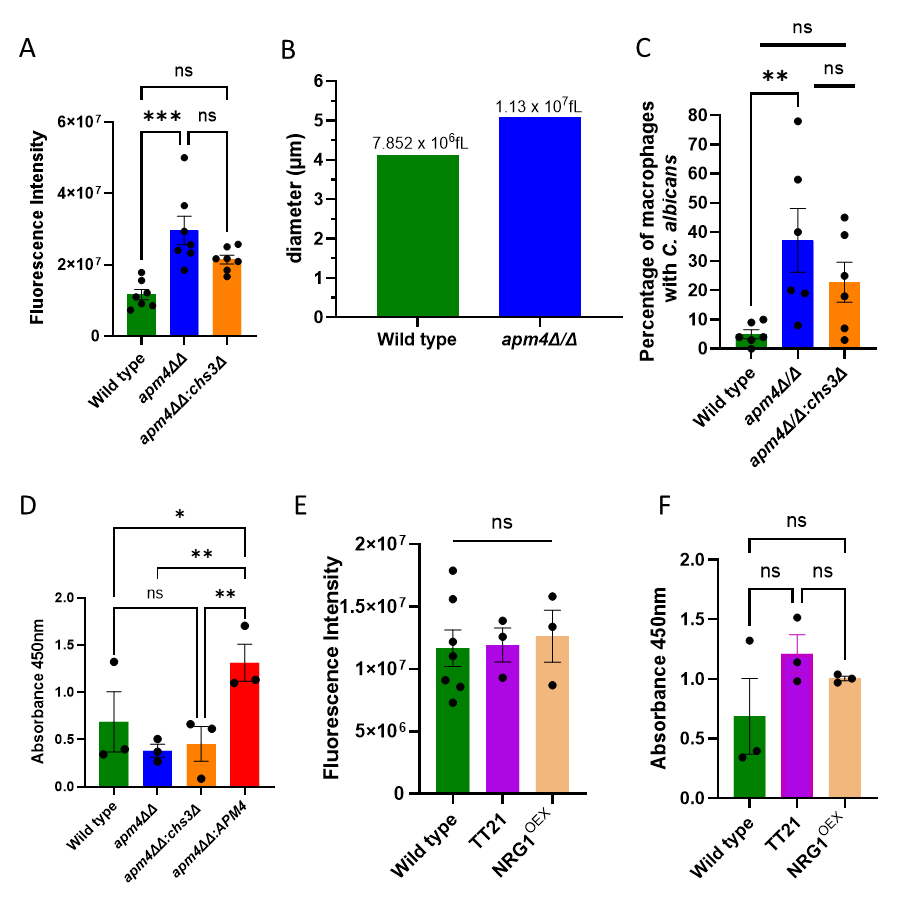


**Supplementary Figure 1**

**The cell biology of cells that lack AP-2.** A) The CASY counter was used to automatically count the cell size and volume of overnight cultures refreshed for 30 minutes at 30^o^C the n =>200000. The graph presents the mean diameter of cells, the numbers represent the average volume of cells in fL. B) C. albicans overnight cultures were incubated with fresh media for 3 hours before staining with Calcofluor white and imaging with a fluorescent microscope. The dataset from 3 biological replicates is presented in the two graphs where the integrated intensity of Calcofluor white staining was plotted. The data were analysed using a Mann-Whitney test of individual cell fluorescence values. C) The proportion of macrophages in a field of view that phagocytose C. albicans were quantified. Macrophages were infected with an MOI of 1 by C. albicans. 5 independent replicates with approximately 6 images per replicate. Each circle represent the average of each replicate analyzed. Mann-Whitney statistical tests was performed using values from individual images. D) XTT metabolic assay of C. albicans cells grown in plastic 96 well plates for 24 hours, the results are from 3 independent biological replicates were collected at absorbance 450nm. One-way ANOVA statistical tests was used. E) C. albicans overnight cultures were incubated with fresh media for 3 hours before staining with Calcofluor white and imaging with a fluorescent microscope. The dataset from 3 biological replicates is presented in the two graphs where the integrated intensity of Calcofluor white staining was plotted. The data were analysed using a Mann-Whitney test of individual cell fluorescence values. F) XTT metabolic assay of C. albicans cells grown in plastic 96 well plates for 24 hours, the results are from 3 independent biological replicates were collected at absorbance 450nm. One-way ANOVA statistical tests was used. . ns P< 0.05; * P=0.05-0.01; ** P= 0.01-0.001; *** P = 0.001-0.0001; **** P ≤ 0.0001.
